# Twist1 Inactivation in Dmp1-Expressing Cells Increases Bone Mass but Does Not Affect the Anabolic Response to Sclerostin Neutralization

**DOI:** 10.3390/ijms20184427

**Published:** 2019-09-09

**Authors:** Karl J. Lewis, Roy B-J Choi, Emily Z. Pemberton, Whitney A. Bullock, Anthony B. Firulli, Alexander G. Robling

**Affiliations:** 1 Department of Anatomy & Cell Biology, Indiana University School of Medicine, Indianapolis, IN 46202, USA; 2 Department of Pediatrics, Indiana University School of Medicine, Indianapolis, IN 46202, USA; 3 Herman B. Wells Center for Pediatric Research, Indiana University School of Medicine, Indianapolis, IN 46202, USA; 4 Department of Biomedical Engineering, Indiana University–Purdue University at Indianapolis, Indianapolis, IN 46202, USA; 5 Indiana Center for Musculoskeletal Health, Indianapolis, IN 46202, USA; 6 Richard L. Roudebush VA Medical Center, Indianapolis, IN 46202, USA

**Keywords:** Twist1, sclerostin, Wnt, osteoporosis, osteocytes, mechanotransduction

## Abstract

Wnt signaling plays a major role in bone metabolism. Advances in our understanding of secreted regulators of Wnt have yielded several therapeutic targets to stimulate osteoanabolism—the most promising of which is the Wnt inhibitor sclerostin. Sclerostin antibody recently gained approval for clinical use to treat osteoporosis, but the biology surrounding sclerostin antagonism is still incompletely understood. Numerous factors regulate the efficacy of sclerostin inhibition on bone formation, a process known as self-regulation. In previous communications we reported that the basic helix-loop-helix transcription factor Twist1—a gene know to regulate skeletal development—is highly upregulated among the osteocyte cell population in mice treated with sclerostin antibody. In this communication, we tested the hypothesis that preventing Twist1 upregulation by deletion of Twist1 from late-stage osteoblasts and osteocytes would increase the efficacy of sclerostin antibody treatment, since Twist1 is known to restrain osteoblast activity in many models. Twist1-floxed loss-of-function mice were crossed to the Dmp1-Cre driver to delete Twist1 in Dmp1-expressing cells. Conditional Twist1 deletion was associated with a mild but significant increase in bone mass, as assessed by dual energy x-ray absorptiometry (DXA) and microCT (µCT) for many endpoints in both male and female mice. Biomechanical properties of the femur were not affected by conditional mutation of Twist1. Sclerostin antibody improved all bone properties significantly, regardless of Twist1 status, sex, or endpoint examined. No interactions were detected when Twist1 status and antibody treatment were examined together, suggesting that Twist1 upregulation in the osteocyte population is not an endogenous mechanism that restrains the osteoanabolic effect of sclerostin antibody treatment. In summary, Twist1 inhibition in the late-stage osteoblast/osteocyte increases bone mass but does not affect the anabolic response to sclerostin neutralization.

## 1. Introduction

The search for osteoanabolic targets in bone tissue has produced few clinically viable candidates despite the paucity of, and need for, anabolic therapies to treat a variety of bone diseases [1]. Until very recently, only two anabolic agents were approved for clinical use (teriparatide and abaloparatide), both of which use a common mechanism to achieve increased bone formation—stimulation of the parathyroid hormone/parathyroid hormone-related receptor PTH1R [2]. These therapies require daily injections and are efficacious only for around 18 months of treatment. Other options for stimulating new bone formation are needed.

A greater understanding of the Wnt signaling pathway in the context of bone tissue has revealed new opportunities for building bone in the skeleton. The rationale for targeting Wnt components to improve bone properties comes from several human mutations that result in increased bone mass, including loss-of-function mutations in the SOST gene (e.g., sclerosteosis) [3], loss-of-function mutations in the LRP4 gene (e.g., sclerosteosis type 2) [4], and gain-of-function mutations in the LRP5 gene (e.g., endosteal hyperostosis, or high bone mass (HBM) disease) [5]. Sclerostin, the secreted inhibitor of Wnt–Lrp5/6 signaling (encoded by the SOST gene), has gained the most traction as a target to increase bone mass for reasons that include high bone specificity of the protein, very high potency (as revealed by the severe osteosclerotic phenotype among patients that fail to synthesize sclerostin), and the fact that sclerostin is a secreted protein that interacts extracellularly, which renders it amenable to targeting with biologics [6]. Neutralizing antibodies to sclerostin have had great success in phase III trials for postmenopausal osteoporosis [7], and a sclerostin neutralizing antibody—Romosozumab—was recently approved for clinical use in Japan, the US, South Korea, Canada, and Australia [8].

While sclerostin neutralization is unequivocally efficacious in stimulating new bone formation, the mechanisms of action and associated biology are less clear. We and others have reported that sclerostin inhibition triggers the expression of other Wnt inhibitors (e.g., Dkk1) that ultimately limit the potency of sclerostin neutralization [9,10]. Disabling some of the accessory Wnt inhibitory mechanisms can produce a synergistic effect on bone formation that is well beyond the individual or even additive effects of Wnt inhibitors. We and others have also reported that many genes exhibit changes in expression level in response to sclerostin neutralization [11,12,13]. One of the most consistent and most highly upregulated transcripts in osteocytes among mice exposed to sclerostin antibody is the basic helix-loop-helix (bHLH) transcription factor Twist1 [11,13]. Twist1 haploinsufficiency in humans causes Saethre-Chotzen syndrome, characterized by premature fusion of the cranial vault bones and distal limb skeletal deformities, among other developmental defects [14]. Twist1 overexpression inhibits osteoblast differentiation in vivo [15] and in vitro [16], whereas Twist1 knockdown increases osteoblast differentiation and mineralization in vitro [17]. Thus, we surmised that Twist1 upregulation in sclerostin-deprived osteocytes (e.g., in antibody-treated mice) might be another self-regulation mechanism that limits the otherwise fuller anabolic potential of sclerostin neutralization. We studied the skeletal phenotype of mice with conditional deletion of Twist1 in the late-stage osteoblast/osteocyte population using Dentin Matrix Protein-1 Cre (^10kb^Dmp1-Cre) to recombine homozygous Twist1 loss-of-function alleles (Twist1^f/f^). Further, we tested the efficacy of sclerostin-neutralizing antibody in the conditional mice, which were unable to upregulate Twist1 in response to antibody treatment. We found that although the conditional mutant mice had increased bone mass, sclerostin antibody treatment resulted in equivalent gains in bone mass regardless of osteocytic Twist1 status. Therefore, it is unlikely that increased Twist1 expression in the osteocyte limits the efficacy of sclerostin neutralization.

## 2. Results

### 2.1. Twist1 Induction in Scl-Ab-Treated Mice

**Figure 1:**
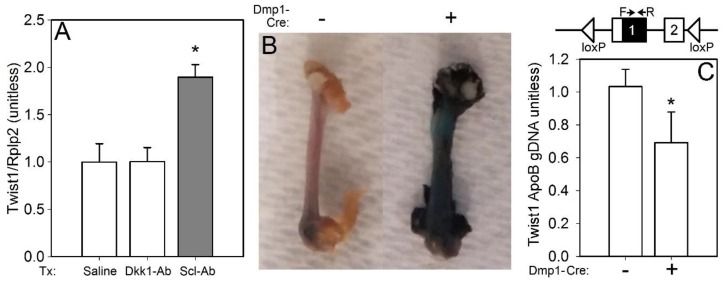
(**A**) Twist1 expression, normalized to the housekeeper Rplp2, was significantly increased in osteocyte-enriched bone tissue lysates from 10-wk-old female WT C57Bl/6J mice that were injected with a single dose of 25 mg/kg sclerostin antibody (Scl-Ab), then sacrificed 2 days later. The same procedure using Dkk1 antibody (25 mg/kg) did not change Twist1 expression; *n* = 4/group, * *p* < 0.05. (**B**) Representative micrographs of β-galactosidase-reacted femora from Twist1^f/f^ mice that also carried the Rosa26-LacZ Cre reporter line. Qualitatively, negligible lacZ staining is apparent in the Cre-negative mice, but the mice positive for ^10kb^Dmp1-Cre exhibit a strong reaction in the bone tissue, indicating recombination activity of the Cre transgene. (**C**) Top: schematic showing the floxed Twist1 locus (2 exons) and the location within exon 1 of the forward and reverse primers (arrows) used to probe for the intact floxed allele in genomic DNA from cortical bone. Bottom: ratio of intact Twist1 sequence (target) to Apolipoprotein B sequence (control), in Cre-positive and Cre-negative mice; *n* = 8–9/group, * *p* < 0.05.

We have previously reported a significant increase in Twist1 expression in sclerostin antibody (Scl-Ab)-treated mice, based on high-throughput sequencing of the osteocyte transcriptome [11]. To repeat and validate this result in a more quantitative assay, we treated WT mice with a single injection of Scl-Ab and measured Twist1 expression in the long bone cortices via qPCR 2 days later. In addition, to address whether Twist1 regulation might be generally Wnt responsive (e.g., also responsive to suppression of other Wnt inhibitors) in bone, or if its regulation is more selective compared to other Wnt inhibitors, a Dkk1 antibody (Dkk1-Ab) group was included as a separate treatment. Twist1 was significantly upregulated by Scl-Ab but not by Dkk1-Ab, as compared with the saline injection (Figure 1A).

To study Twist1 deficiency in the osteocyte, while avoiding lethality and/or developmental defects that arise from global mutant alleles (e.g., null [18], hypomorphic [19]) or from conditional alleles targeted to early stage mesenchymal cells (e.g., Prx1-Cre × Twist1 flox), we crossed the ^10kb^Dmp1-Cre driver onto a Twist1^f/f^ background. Inclusion of the Rosa26-LacZ reporter allele in the breeding scheme revealed recombination in bone, as indicated by strong β-galactosidase staining in ^10kb^Dmp1-Cre-positive (but not negative) femora (Figure 1B). Further, no limb patterning defects were found in the Cre-positive mice (data not shown), which was consistent with Dmp1 expression in the late-stage mesenchymal-lineage cell (e.g., late-stage osteoblast and osteocyte). Cortical bone genomic DNA was assayed for the intact Twist1 allele, which was found in ~60% of the Cre-positive extracts, compared to Cre-negative extracts (Figure 1C).

### 2.2. Mice with Loss-of-Function Twist1 Alleles in Dmp1-Expressing Cells Have a Late-Onset Increase in Bone Mineral Density, with Similar Response to Sclerostin Neutralization as Control Mice

**Figure 2:**
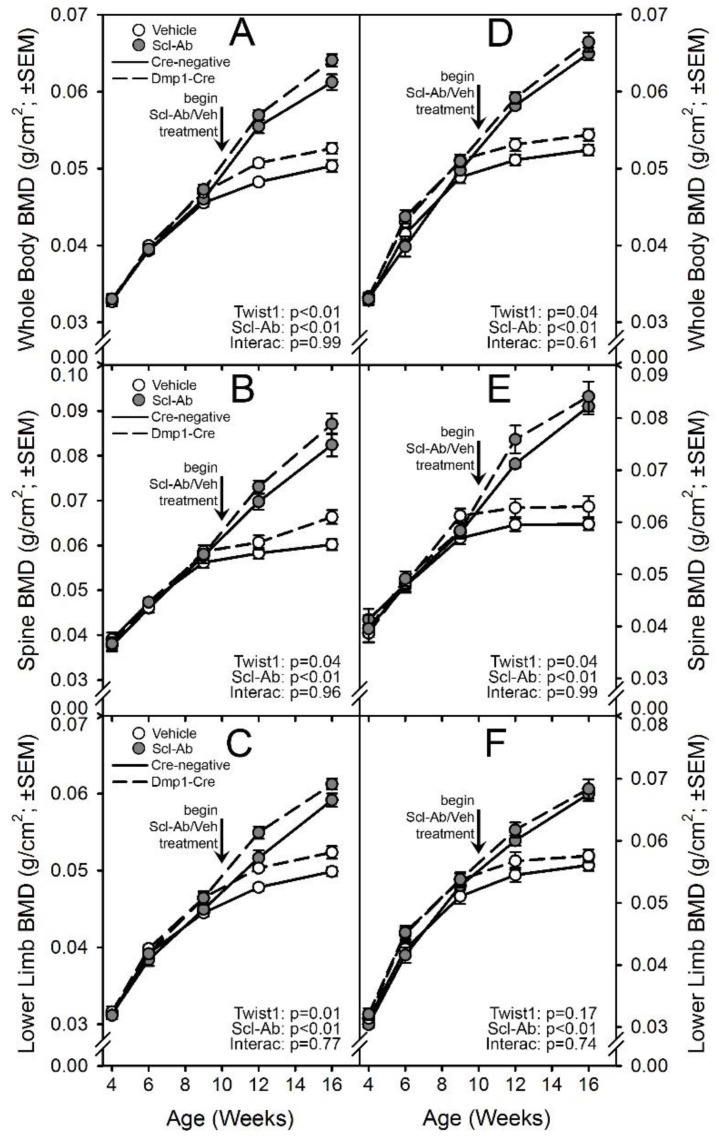
Serial in vivo DXA scans of Cre-negative (solid lines) and ^10kb^Dmp1-Cre-positive (broken lines) Twist1^f/f^ mice, treated twice per week with vehicle control (open circles) or 25 mg/kg sclerostin antibody (Scl-Ab; filled circles). Scans were collected every 2–4 wk and analyzed for (**A**,**D**) whole-body BMD, (**B**,**E**) lumbar spine BMD, and (**C**,**F**) BMD of the right hindlimb distal to the acetabulum. Panels A–C display data from female mice; panels D–F display data from male mice. Antibody/vehicle treatment began at 10 wk of age, indicated by the vertical arrow. The longitudinal data were tested for significance of both main effects and an interaction using rmANOVA, reported in the corner of each panel; *n* = 8–11/group.

To determine the skeletal effects of late-stage Twist1 deletion in bone, changes in bone mineral density (BMD) among mice with ^10kb^Dmp1-Cre-driven inactivation of Twist1 were compared to those of Cre-negative mice by considering only the vehicle-treated groups. Serial whole-body DEXA scans were collected from all experimental mice intermittently from 4 to 16 wk of age. Repeated measures ANOVA using all time points collected indicated that Cre-positive mice had significantly increased BMD only for the whole-body ROI in males, and the lower-limb ROI in females (Figure 2). However, when just the later time points (beyond 6 wk of age) were analyzed, significant increases in BMD were found among the Cre-positive mice for all ROIs examined with the exception of the lower-limb ROI among males. Body weight was not different among males, but female Cre-positive mice were significantly heavier than Cre-negative mice (Figure S1).

Five weeks of treatment with Scl-Ab significantly increased BMD at all ROIs, in both sexes, in both Twist1 replete (Cre-negative) and Twist1 compromised (Cre-positive) mice. By the end of the experiment, BMD was 17–20% greater in the lower limb, 31–38% greater in the spine, and 22–24% greater for the whole body among Scl-Ab-treated mice compared to the genotype/sex-matched vehicle controls. Two-way ANOVA failed to identify a significant interaction term for any of the DXA outcome variables, suggesting that the Twist1 bone deletion did not significantly change the influence of Scl-Ab on densitometric properties.

### 2.3. Twist1 Conditional Mutant Mice Have Improved Cortical Bone Properties and Sex-Specific Changes in Cancellous Bone, but Have Similar Architectural Response to Scl-Ab as Control Mice

**Figure 3:**
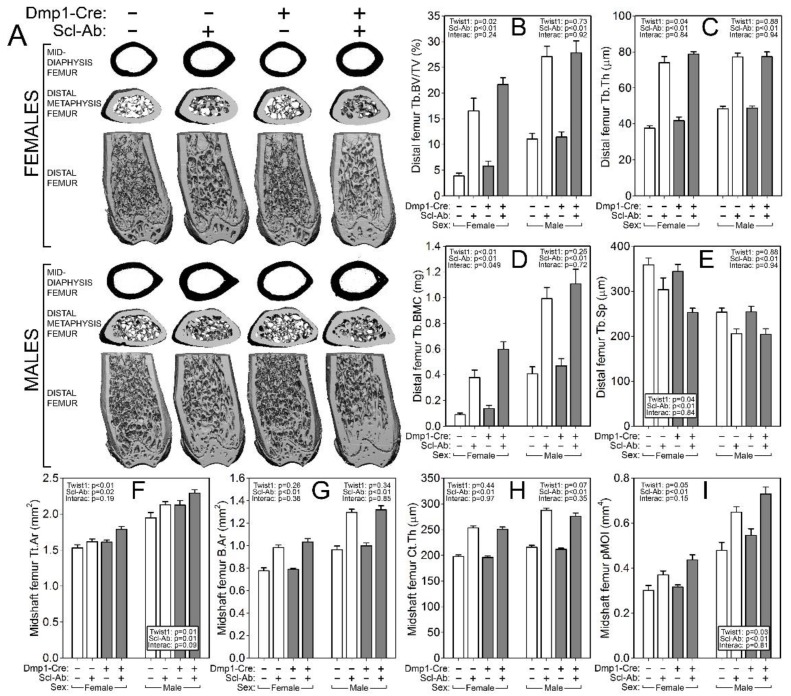
µCT-derived measurement of the distal femur metaphyseal cancellous bone and mid-femur cortical bone from Cre-negative (white bars) and ^10kb^Dmp1-Cre-positive (grey bars) Twist1^f/f^ mice at 16 wk of age, treated with or without sclerostin antibody (Scl-Ab) for 6 wk prior to sacrifice. (**A**) Representative 3D reconstructions of (top row) the midshaft femur, (middle row) the distal metaphysis (proximal view), and (bottom row) the caudal half of the distal femur (the ventral half was digitally removed). Female mice are in the top of the panel, males in the bottom. Quantitative differences in (**B**) femur trabecular bone volume fraction (BV/TV), (**C**) trabecular thickness (Tb.Th), (**D**) trabecular bone mineral content (Tb.BMC), and (**E**) trabecular separation (Tb.Sp) are shown for female (left side of each panel) and male (right side of each panel) mice. Quantitative differences in (**F**) femur cortical bone total area (Tt.Ar), (**G**) bone area (B.Ar), (**H**) cortical thickness (Ct.Th), and (**I**) polar moment of inertia (pMOI) are shown for female (left side of each panel) and male (right side of each panel) mice. Data were tested for significance using two-way ANOVA within sex, with Twist1 status and antibody as the main effects (plus interaction), which are reported in the corner or base of each sex grouping; *n* = 8–11/group.

**Figure 4:**
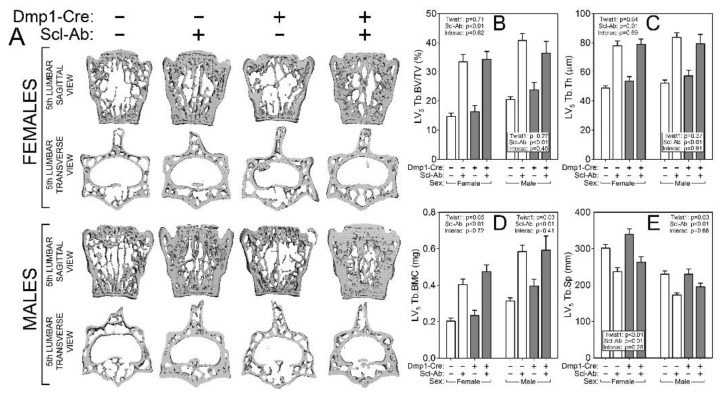
µCT-derived measurement of the fifth lumbar vertebral body cancellous bone from Cre-negative (white bars) and ^10kb^Dmp1-Cre-positive (grey bars) Twist1^f/f^ mice at 16 wk of age, treated with or without sclerostin antibody (Scl-Ab) for 6 wk prior to sacrifice. (**A**) Representative 3D cutaway reconstructions of the fifth lumbar vertebra in A-P view (top row) and S-I view (bottom row). Female mice are in the top of the panel, males in the bottom. Quantitative differences in lumbar vertebra 5 (LV_5_) (**B**) trabecular bone volume fraction (BV/TV), (**C**) trabecular thickness (Tb.Th), (**D**) trabecular bone mineral content (Tb.BMC), (**E**) and trabecular separation (Tb.Sp) are shown for female (left side of each panel) and male (right side of each panel) mice. Data were tested for significance using two-way ANOVA within sex, with Twist1 status and antibody as the main effects (plus interaction), which are reported in the corner or base of each sex grouping; *n* = 8–11/group.

**Figure 5:**
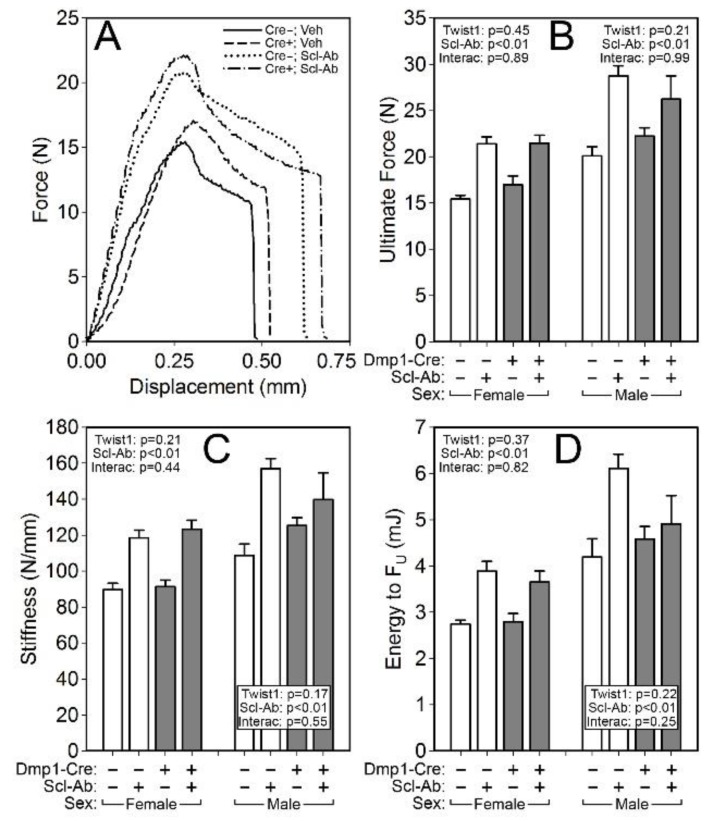
(**A**) Representative force-displacement curves from monotonic three-point bending tests to failure conducted on whole femora from female Cre-negative and ^10kb^Dmp1-Cre-positive Twist1^f/f^ mice at 16 wk of age, treated with or without sclerostin antibody (Scl-Ab) for 6 wk prior to sacrifice. Quantification of (**B**) ultimate force, (**C**) stiffness, and (**D**) energy to ultimate force revealed strong antibody effects in both Twist-replete and -deficient mice, but no effect of the Twist mutation alone was detected. Data were tested for significance using two-way ANOVA within sex, with Twist1 status and antibody as the main effects (plus interaction), which are reported in the corner or base of each sex grouping; *n* = 8–11/group.

To probe compartment-specific effects of both the Twist1 mutation alone and the response to Scl-Ab in different Twist1 backgrounds, the femur and spine were scanned via µCT to collect distal femur and L_5_ cancellous properties and midshaft femur cortical properties. Bone volume fraction (BV/TV), trabecular thickness and spacing (Tb.Th and Tb.Sp) and bone mineral content (BMC) were significantly improved by the Twist1 mutation in female but not male mice (Figure 3). No changes in spine cancellous properties were detected in Twist1 mutants as compared to controls for either sex (Figure 4). However, cortical bone was improved by Twist1 deletion in both male and female mice, as indicated by a significant genotype effect for midshaft femur total area (Tt.Ar) and polar moment (pMOI) (Figure 3). The increase in µCT-derived cortical properties prompted us to test the cortical bone for biomechanical changes, but no significant genotype-related changes in ultimate force, stiffness, or energy absorption were found (Figure 5).

Similar to the results reported for DXA measurements, the µCT measurements and mechanical testing revealed a strong effect of Scl-Ab treatment for every endpoint analyzed, but no significant interaction terms were found for genotype by treatment, suggesting that the Twist1 bone deletion did not significantly change the influence of Scl-Ab on cancellous or cortical properties.

## 3. Discussion

Our goal in this study was to determine whether preventing Twist1 upregulation during sclerostin inhibition could improve the efficacy of sclerostin antibody therapy. That goal was prompted in part by our previously published RNAseq-derived data showing a 2.6-fold upregulation in Twist1 expression in osteocyte-enriched lysates from Scl-Ab-treated mice as compared with saline-treated mice [11]. Similarly, the Amgen group reported that Twist1 was among the most highly upregulated genes in osteocyte-enriched lysates from rats exposed to Scl-Ab, reaching a three-fold increase by 7 days post Scl-Ab injection [20]. Scl-Ab is known to induce self-regulating mechanisms on Wnt signaling, including Sost and Dkk1 expression [9], which might serve to limit excessive bone formation. We surmised that Twist1 might be a component of the self-regulating mechanism of Scl-Ab, given that Twist1 suppresses osteogenesis and mineralization in culture [21]. Treatment with Scl-Ab was equally efficacious in the skeletons of Twist1 mutants and controls, suggesting that upregulation of Twist1 is not a functional compensatory mechanism that restrains the bone-building effects of sclerostin neutralization. It is possible that deletion of Twist1 earlier in the MSC/osteoblast lineage would have more profound effects on Scl-Ab efficacy. It is noteworthy that we observed an increase in Twist1 expression with Scl-Ab but not Dkk1-Ab injection; we have previously reported that longer-term Scl-Ab (but not Dkk1-Ab) treatment is anabolic in bone.^9^ Nevertheless, Twist1 inhibition is unlikely to improve Scl-Ab efficacy in patients, despite its baseline effect on bone homeostasis.

A second goal of this study was to learn whether late-stage deletion of Twist1 induces skeletal effects that enhance bone mass, while avoiding development and patterning defects normally associated with Twist1 mutations. We found that Twist1 deletion from Dmp1-expressing cells resulted in a mild but significant increase in bone mass, particularly in older (beyond 6 wk) mice and preferentially in the cortical bone compartment; increased cancellous bone properties were observed only in female mutants. The significant increase in midshaft femur size (Tt.Ar) and estimated torsional rigidity (pMOI) observed among Cre-positive mice did not translate into improved mechanical competence of the bones, though there was a trend of increased strength among Cre-positive mice.

In contrast to our finding that Twist1 expression increases in mice where Wnt signaling is elevated by sclerostin neutralization, tonic activation of the same pathway in osteoblasts from Lrp5-HBM patients appears to reduce Twist1 expression compared to controls [22]. The difference in Twist1 dynamics might be related to species differences, or to a chronic versus acute stimulation of Wnt signaling in bone. It is also possible that since recombination in the osteocyte population was incomplete (Figure 1C), perhaps antibody treatment was able to upregulate Twist expression among osteocytes with unrecombined alleles. In addition, we have previously reported that sclerostin expression is downregulated by mechanical loading of osteocytes [23]. In the same cell population, loading induces a significant increase in Twist1 expression that is temporally preceded by Sost downregulation [24]. Whether Twist1 plays a role in mechanical signaling remains to be determined.

The role of Twist1 in postnatal bone has not been widely studied, and where it has, its role in mineralized tissue homeostasis is equivocal. For example, PTH has been reported to both increase [25] and decrease [26] Twist1 expression. Twist1 haploinsufficiency in mice is reported to improve skeletal healing and osteogenesis around defects [27], but other reports indicate it reduces bone formation [28]. Moreover, Twist1 overexpression promotes odontoblast differentiation [29] but impairs cementoblast differentiation [30]. As a widely expressed transcription factor, the role of Twist1 in regulating postnatal skeletal cell fate and activity is likely to be highly context-dependent. Twist1 is a candidate target for non-small cell lung cancer therapy [31], and development of small molecule inhibitors of Twist1 is an area of great interest [32]. If a Twist1 inhibitor becomes clinically available, our data indicate that it is unlikely to have negative effects on bone tissue. Moreover, Twist1 inhibition is unlikely to interfere with the anabolic effects of Romosozumab therapy.

One potential limitation of our experiments is that they were conducted entirely in in vivo models. There is a paucity of information regarding the general function of Twist1 in osteocytes, which could be addressed using cell culture approaches involving Twist1 knockdown or overexpression in osteocytes. However, it is difficult to study sclerostin-mediated osteocyte biology in culture. Many of the common osteocyte cell lines express sclerostin at very low levels and thus antibody-based inhibition of such a scarce target is challenging. For this reason, we pursued the experiments using an in vivo model where osteocyte-selective Twist1 regulation of skeletal physiology, particularly in response to sclerostin antibody, could be studied.

In summary, deletion of Twist1 from the osteocyte/late-stage osteoblast population resulted in increased bone mass and density, which was more robustly manifested in young adult and adult mice. Treatment with sclerostin antibody was equally efficacious in enhancing bone mass, size, and strength in Twist1 mutants and WT mice. While elucidation of the self-regulation phenomenon associated with sclerostin inhibition is an area of great interest, Twist1 transcriptional activity does not appear to be among those factors that induce restraint on the effects of sclerostin neutralization in bone.

## 4. Materials and Methods

### 4.1. Mice

Development of the conditional Twist1-flox mouse model has been reported elsewhere [33]. Briefly, the Twist1-flox allele harbors head-to-toe loxP sequences flanking exons 1 and 2 of the endogenous Twist1 gene. Recombination of the loxP sites results in complete deletion of the entire open reading frame, which is contained in exon 1. The Rosa26-LacZ Cre-reporter line (B6.129S4-*Gt(ROSA)26Sor^tm1Sor^*/J) has been described elsewhere [34]. Briefly, these mice harbor a loxP-flanked DNA STOP sequence preventing expression of the downstream lacZ cassette. When crossed with a Cre transgenic strain, the stop sequence is removed and lacZ is expressed in cells/tissues where Cre is expressed. Development of the ^10kb^Dmp1-Cre transgenic mouse model has been reported elsewhere [35]. Briefly, these mice harbor a transgene expressing Cre recombinase driven by a 9.6kb fragment of the mouse Dentin Matrix Protein-1 promoter. All mouse colonies were maintained on a C57BL/6 background. For all experiments, Cre-negative Twist1^f/f^ littermates were used as controls. All mouse procedures were performed in accordance with the IACUC guidelines and approvals (approval date: 11/17/2017).

### 4.2. Antibodies 

Details of the development of sclerostin- and Dkk1-neutralizing antibodies has been reported elsewhere [36,37]. Briefly, the sclerostin antibody (Scl-Ab) is a ratized version of a mouse monoclonal antibody that neutralizes mouse sclerostin. The Dkk1 antibody is a neutralizing rat monoclonal raised against mouse Dkk1. For the functional study, Scl-Ab was injected into mice subcutaneously (subQ) at 25 mg/kg, 200 µL per injection, twice per week for 5 wk, beginning at 10 wk of age. Vehicle treatment was the saline buffer in which the antibodies were made, and was injected with identical frequency, volume, and duration as described for antibody injections. For short-term gene expression studies, either Scl-Ab or Dkk1-Ab were injected once at 25 mg/kg each into 10-wk-old mice, and the mice were sacrificed 2 days later.

### 4.3. Dual-Energy X-Ray Absorptiometry (DXA)

Collection of serial DXA measurements on live mice are described and validated elsewhere [11]. Briefly, mice were anesthetized via inhalation of 2.5% isoflurane (IsoFlo; Abbott Laboratories, North Chicago, IL) mixed with O_2_ (1.5 liter/min) for a total ~8 min, including both induction and scanning. The mice were placed in prone position on a specimen tray within the scanner. Whole-body scans were analyzed regionally using the Lunar region of interest (ROI) tools. The ROI for the spine included the third (LV_3_) through fifth (LV_5_) lumbar vertebrae. The ROI for the hindlimb included all skeletal tissue distal the acetabulum. The ROI for the whole body included all skeletal tissues caudal to the CV_1_/skull boundary. Serial scans were performed at 4, 6, 9, 12, and 16 wk of age. Bone mineral density (BMD) was measured for each ROI scan.

### 4.4. Micro-Computed Tomography (μCT)

Formalin-fixed femora and fifth lumbar vertebrae were scanned, reconstructed, and analyzed as previously described [11]. Briefly, 10-μm resolution, 50-kV peak tube potential and 151-ms integration time were used to collect scans on a Scanco uCT-35 tomographer. The distal 60% of each femur and the entire body of each vertebra were scanned. Standard parameters related to cancellous and cortical bone architecture were measured [38].

### 4.5. β-Galactosidase Staining, Twist1 Expression, and Twist1 Genomic Recombination

Whole femora were removed from 8-wk-old ^10kb^Dmp1-Cre-positive and -negative mice that also carried the LacZ reporter. Femora were fixed overnight in 0.5% glutaraldehyde, then reacted for 1 h in β-gal staining solution (1 mg/mL X-gal, 5 mM potassium ferricyanide, 5 mM potassium ferrocyanide) at 37 °C. Samples were rinsed in tap water and photographed. Twist1 RNA expression was measured in pulverized femur and tibia cortices from 8-wk-old C57BL/6J mice that had been treated with antibody. Total RNA was isolated using the Trizol method and reverse transcribed into cDNA using random hexamers. Twist1 and Rplp2 (housekeeping transcript) were measured using the SYBR green technique on an ABI QuantStudio 7 Flex thermocycler. The ΔΔCT method was used to calculate relative expression, which was normalized to Rplp2 for each sample. Recombination of the floxed allele was assessed by real-time PCR of purified genomic DNA (gDNA) extracted from cortical bone tissue from the distal tibia. Both primers were located within the floxed region (Figure 1C). Recombination of Twist 1 gDNA was standardized to Apolipoprotein B gDNA.

### 4.6. Mechanical Properties

Parameters related to whole-bone strength were measured using three-point bending tests on isolated femora, as previously described [39]. Briefly, each femur was loaded to failure in monotonic compression, during which force and displacement were collected every 0.01 s. From the force/displacement curves, ultimate force, stiffness, and energy to failure were calculated using standard equations [40].

### 4.7. Statistics

Statistical analyses were computed with JMP (version 12.0, SAS Institute Inc., Cary, North Carolina, USA). The tomography and biomechanical endpoints were analyzed with two-way ANOVA within sex, using Cre status and injection treatment as the main effects. When at least one main effect was significant, interaction terms were calculated and tested for significance. Time-series data were analyzed with repeated-measures ANOVA. Statistical significance was taken at *p* < 0.05. Two-tailed distributions were used for all analyses. Data are presented as mean ± SEM. A minimum of eight animals were included in each group (*n* = 8–11/group).

## Supplementary Materials

Supplementary materials can be found at https://www.mdpi.com/1422-0067/20/18/4427/s1.
