# Supplementary material for: Twist1 Inactivation in Dmp1-Expressing Cells Increases Bone Mass but Does Not Affect the Anabolic Response to Sclerostin Neutralization
Source: Int J Mol Sci. 2019 Sep 9;20(18):4427. doi: 10.3390/ijms20184427 (PMC6769567; doi:10.3390/ijms20184427)
Supplement: Supplementary file 1 [file ijms-20-04427-s001.pdf]

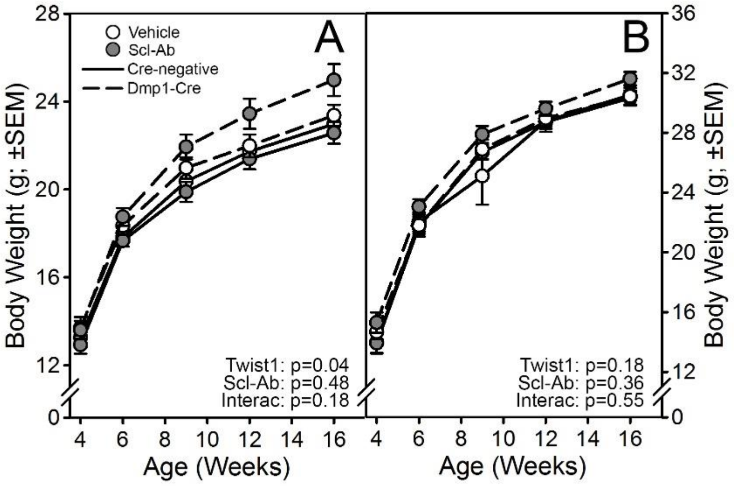

Figure S1. Body mass in Cre-negative (solid lines) and 10kbDmp1-Cre positive (broken lines) Twist1f/f mice, treated twice per week with vehicle control (open circles) or 25 mg/kg sclerostin antibody (Scl-Ab; filled circles). Body mass was measured every 2–4 wks in both (**A**) female and (**B**) male mice. The longitudinal weight data were tested for significance of both main effects and an interaction using rmANOVA, reported in the corner of both panels. *N* = 8–11/group.
